# Selective electrodeposition of indium microstructures on silicon and their conversion into InAs and InSb semiconductors

**DOI:** 10.1186/s11671-023-03778-9

**Published:** 2023-02-07

**Authors:** Katarzyna E. Hnida-Gut, Marilyne Sousa, Preksha Tiwari, Heinz Schmid

**Affiliations:** 1 IBM Research Europe – Zurich, Zurich, Switzerland; 2 IHP Leibniz Institute for High Performance Microelektronics, Frankfurt (Oder), Germany; 3 Polariton Technologies Ltd., Zurich, Switzerland

**Keywords:** Integration, Saturation, Electrodeposition, Recrystallization, III-Vs, TASE

## Abstract

**Abstract:**

The idea of benefitting from the properties of III-V semiconductors and silicon on the same substrate has been occupying the minds of scientists for several years. Although the principle of III-V integration on a silicon-based platform is simple, it is often challenging to perform due to demanding requirements for sample preparation rising from a mismatch in physical properties between those semiconductor groups (e.g. different lattice constants and thermal expansion coefficients), high cost of device-grade materials formation and their post-processing. In this paper, we demonstrate the deposition of group-III metal and III-V semiconductors in microfabricated template structures on silicon as a strategy for heterogeneous device integration on Si. The metal (indium) is selectively electrodeposited in a 2-electrode galvanostatic configuration with the working electrode (WE) located in each template, resulting in well-defined In structures of high purity. The semiconductors InAs and InSb are obtained by vapour phase diffusion of the corresponding group-V element (As, Sb) into the liquified In confined in the template. We discuss in detail the morphological and structural characterization of the synthesized In, InAs and InSb crystals as well as chemical analysis through scanning electron microscopy (SEM), scanning transmission electron microscopy (TEM/STEM), and energy-dispersive X-ray spectroscopy (EDX). The proposed integration path combines the advantage of the mature top-down lithography technology to define device geometries and employs economic electrodeposition (ED) and vapour phase processes to directly integrate difficult-to-process materials on a silicon platform.

**Graphical abstract:**

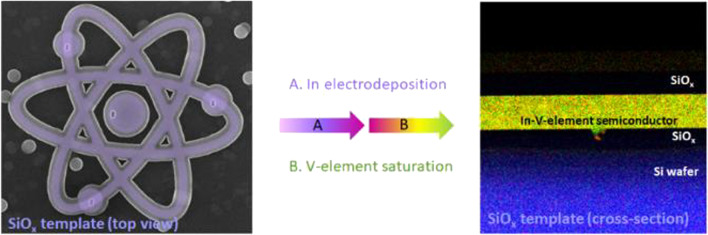

**Supplementary Information:**

The online version contains supplementary material available at 10.1186/s11671-023-03778-9.

## Introduction

Historically, electrodeposition (ED) evolved by creating metal coatings on conductive surfaces for decorative and functional commercial use. As the field has further developed, significant effort was made to extend its use to areas dominated by physical deposition techniques, i.e. microelectronics. However, developing the ED process is an intricate task on its own, and doing this with the idea of compatibility with existing Si-based technologies is another level of difficulty. Although the ED process in most cases allows for low temperatures, low potentials, and relatively simple electrolytes, the necessity of working with conductive substrates limits its applicability. In order to benefit from ED techniques in microelectronic applications, surface modifications are typically applied. Direct ED of metals on semiconductors is not trivial due to this process’s complicated thermodynamics and kinetics, which is influenced by generally low interaction energy between deposited metal atoms and silicon surface. These interactions can be modulated by choosing appropriate ED conditions and avoiding a strongly oxidizing environment [1]. The ED process typically follows the Volmer-Weber growth mechanism, which leads to low growth rates and poor morphology and crystallinity of the deposit. In most cases, electrodeposited material is amorphous/nanocrystalline and further processing is needed to improve its quality. Detailed investigation of gold, copper and platinum deposition mechanisms on silicon can be found in Oskam et al. [1]. Direct electrodeposition of gold [2], copper oxide [3], zinc oxide [4], GaN [5] and recently InSb [6, 7] on Si were shown but are often limited by low adhesion, compatibility issues such as the need for conductive substrates, and non-intentional impurities in the deposits. To resolve the above challenges, careful selection of the electrodeposition process, electrolyte composition and substrate preparation must be made.

This paper focuses on the electrodeposition of In and In-based semiconductors on silicon where literature is sporadic despite its potential for the semiconductor industry. Recently, Neumann et al. published a two-step galvanostatic seed/buffer layer free plating of In on Si using a commercially available electrolyte, demonstrating the feasibility of smooth, continuous films via ED [8]. However, the deposit contained significant amounts of sodium, sulphur, chlorine, and carbon. During our previous research, direct pulse-electrosynthesis of InSb on silicon was developed and similarly significant amounts of carbon and oxygen impurities were observed. The main source of contaminations was by-products from the decomposition of the complexing agents (citric acid and sodium citrate) [6], suggesting that using multicomponent electrolytes might not be the optimum approach for In and In-based semiconductors synthesis. A high purity In deposit is particularly important if it is used as starting material for In- group-V semiconductors. The conversion of In to InP using vapour phosphorization was demonstrated by Ortega and Herrero [9]. In a modified approach, the fabrication of an InP solar cells from electrodeposited or evaporated In on Mo foil [10, 11] via the thin film (vapour–liquid–solid) phosphorization technique (TF-VLS) [12] was shown. E-beam evaporated indium was used for the fabrication of large (> 100 µm) high-mobility InAs and InP crystals at 580 °C and 560 °C, respectively, on a Si/SiO_2_/MoO_x_ substrate [13]. This approach was further modified for the fabrication of optoelectronic grade InP and InAs on Si-based substrates [14–16]. However, the templated liquid phase In process operates in a narrow design window due to geometrical stability constraints of the confined liquid, and no epitaxial relation between crystals and substrates was obtained.

To surpass this limitation, we propose the concept of template-assisted electrodeposition of In directly on a silicon substrate and its saturation with V-element. The process combines features of rapid melt growth in crucibles (RMG) [17, 18] and templated liquid phase growth [15]. The former can achieve epitaxial crystallization of III-Vs on Si but is plagued by unintentional Si doping and Si precipitation stemming from the dissolution of the Si interface during annealing. The latter results in crystals of high purity but having random crystal orientation.

**Fig. 1:**
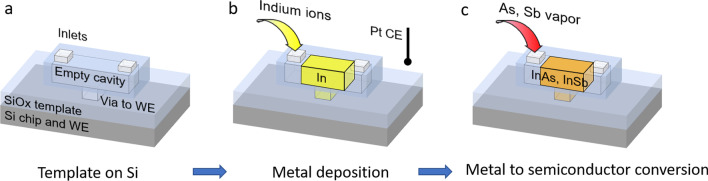
Process flow for selective In-based semiconductor integration on Si. **a** 3D schematic of the template structure. The cavity structure contains inlet holes and a via to the substrate. **b** Indium is electrodeposited from an electrolyte into the cavity using the via to access the WE and a Pt electrode as CE. **c** The In-filled template is transferred and heated in a MOCVD reactor for V-element saturation. Here, the liquid In is converted into a crystalline semiconductor, that nucleates from the Si surface. *WE* working electrode, *CE* counter electrode

The approach (Fig. 1) combines the advantages of fast, uniform template filling characteristics of wet techniques (e.g. electrodeposition) and high-quality semiconductor crystal formation characteristics of vapour phase growth (e.g. metal–organic chemical vapour deposition, MOCVD) methods. It is well known that wet techniques allow to fill different shapes of templates in a short period of time [6]. That means, that even very complicated, nano- or microscale templates can be filled uniformly resulting in an ideal platform for V-element saturation. 3D oxide templates provide a shape to the electrodeposited In (Fig. 1b) and protect it from surface oxidation between the ED and the saturation step. Indium is electrodeposited (Fig. 1b) starting from a small working electrode within the template (region marked as via to WE in Fig. 1a), the template is then placed in an MOCVD chamber, were the liquid In is saturated in the appropriate V-element atmosphere (Fig. 1c), resulting in the synthesis of a III-V semiconductor. In this step, the former working electrode is now acting as a nucleation area for the melt crystallization.

## Results

### Indium electrodeposition in SiO_x_ templates on Si

Indium was selected and electrodeposited because it can be processed with group V elements (P, As, Sb) to form various III-V semiconductors. Furthermore, In is not creating detrimental contamination in contact with Si unlike other metals such as Au or Cu.

Impurities are detrimental to crystallization processes, hampering the formation of monocrystalline materials. To supress the incorporation of contaminants from the electrolyte into the In deposit, we follow a simple yet efficient approach and use only a simple two-component bath: InCl_3_ in DI water with the anticipated In^3+^ reduction reaction (*E* = − 0.34 vs. NHE) (for more details please refer to Experimental section). Therefore, in the unlikely situation of the decomposition of electrolyte the only side-products are gaseous (Cl_2_ and H_2_) and can be extracted from the solution by bubbling with Ar. Another important factor is the choice of the electrochemical technique itself. While more complicated potential-current wave shapes or multistep approaches allow for tuning of the deposit’s composition and morphology [19], they generally require more elaborated electrolytes. Therefore, a 2-electrode chronopotentiometry was used and control of the volume of electrodeposited In was achieved by adjusting the synthesis duration.

**Fig. 2:**
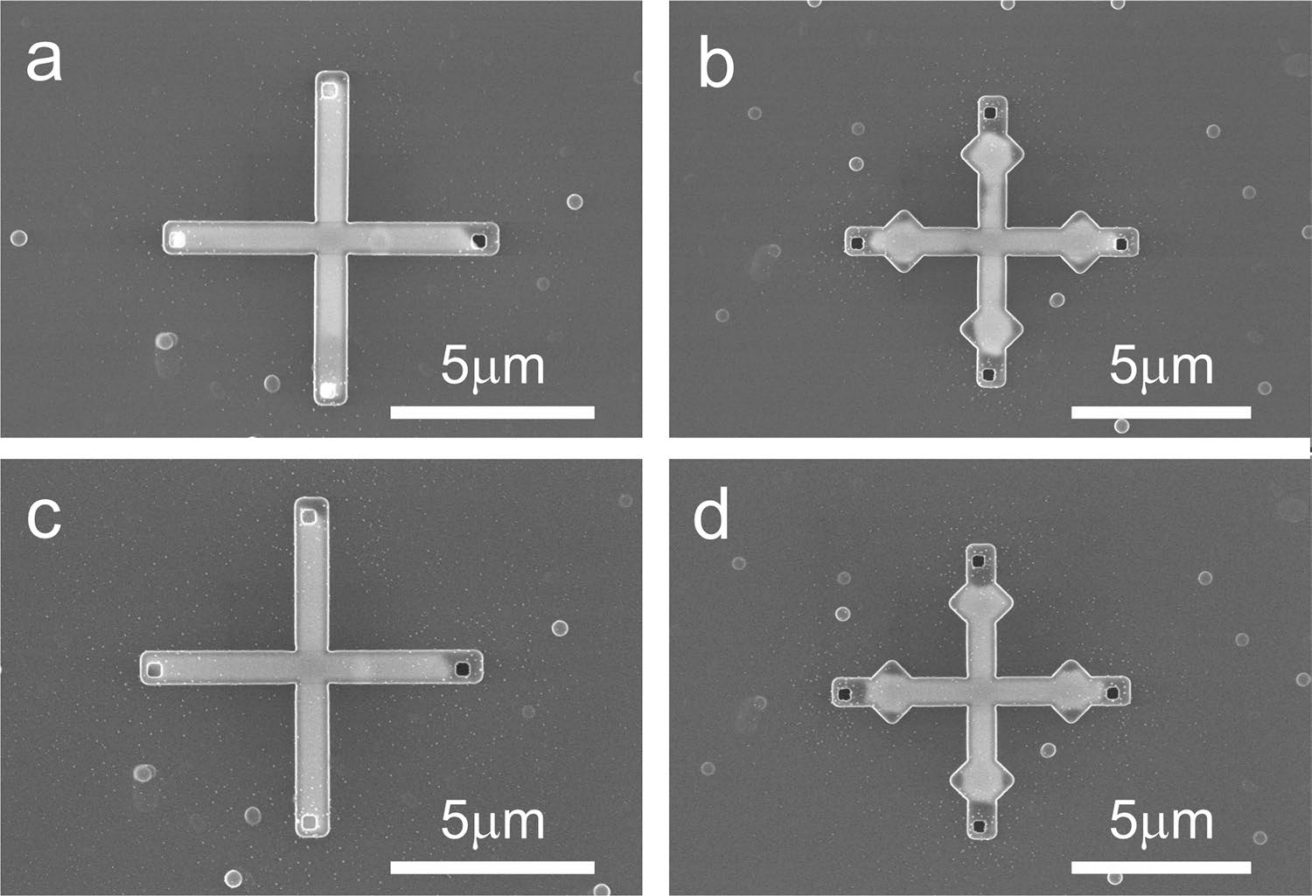
Top-view SEM images of electrodeposited indium inside 300 nm thick templates. Each template has four inlet openings and one (hidden) seed contact to the wafer. **a** The straight legs fill uniformly, while gaps can form at recessed corners **b**. **c**, **d** The same structures as in (**a**, **b**) after melting and solidification. The spherical particles are artefacts from the template fabrication process

Small seed holes embedded in the templates were used as working electrodes. The p-doped Si chips had various template designs of crosses and lines. Figure 2 shows two examples of cross-designs filled with electrodeposited In with the seed (working electrode) contact located at the centre, corresponding to the schematic in Fig. 1a. Overall, the electrodeposition results in uniform filling across the chip. Interestingly, some irregularities in the template filling are observed in designs having recessed corner structures (Fig. 2b). This contrasts with our previous results [6] of conformal filling with amorphous and nanocrystalline InSb deposits.

Free-standing structures made from In are stable up to the melting temperature of *T*_*m*__*In*_ = 157 °C. Beyond that the high surface tension of the melt will lead to the formation of a spherical melt, e.g. droplet. In contrast, the mechanical and three-dimensional confinement by the template should allow for high-temperature processing of the In structure. To verify the mechanical stability of the In-filled SiO_x_ templates beyond *T*_*m*__*In*_ the same device samples were annealed at 560 °C for 30 min in H_2_ (Figs. 2c, d). The samples survived the annealing process despite the high differential expansion between Si/SiO_x_ and the In melt, which is likely a result of the open template structure (presence of the inlets) that allowed for a relaxation of the melt. No visible diffusion or leakage of In through template walls was detected. However, a systematic loss of In close to the inlet holes after annealing is clearly noticeable, which is ascribed to indium out-diffusion through the inlet holes. In addition, the In filling appears slightly more uniform after annealing and solidification. However, some recessed areas of the corner structures remain partially unfilled after solidification.

**Fig. 3:**
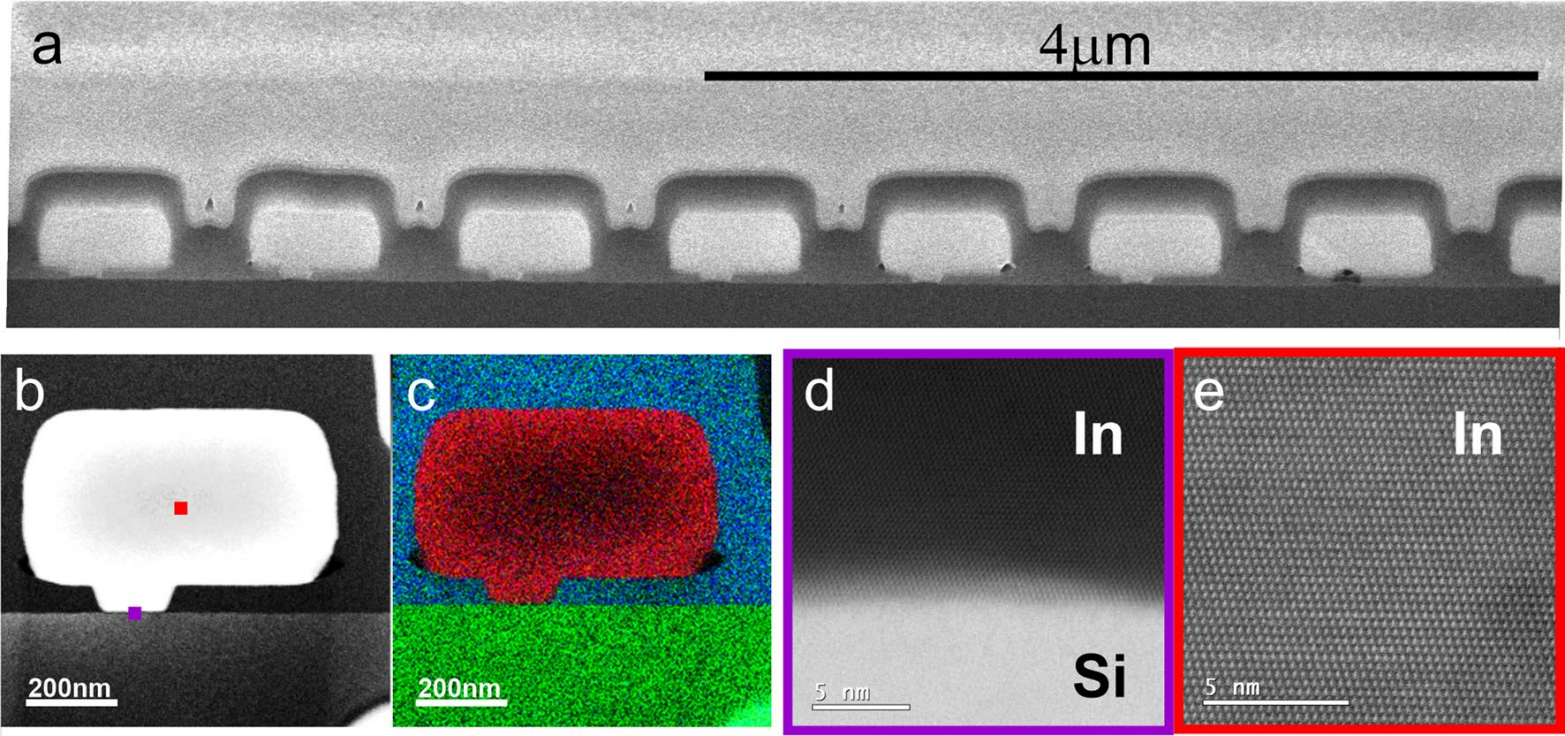
Cross-sectional images of annealed and recrystallized In structures in templates. **a** Low resolution cross section of seven devices. **b** TEM image of a single structure including the seed region. **c** Corresponding EDX map with indium (red), silicon (green) and oxygen (blue). **d**, **e** High-resolution dark field TEM image of In-Si heterointerface and bulk part with collection regions marked with a coloured square in (**b**)

The structure and composition of the annealed samples were analysed by (S)TEM–EDX and are displayed in Fig. 3. Figure 3a shows a low-resolution overview of several device cross sections including seed holes with the In-Si interface. The templates are structurally well preserved, without cracks and remain uniformly filled. A higher magnification image of one of the structures is shown in Fig. 3b. In this sample, areas having a high surface curvature as seen on the bottom corners are sometimes not entirely filled, resulting in localized voids. The elemental analysis of the sample is displayed in the corresponding TEM–EDX map in Fig. 3c, where the red, blue, and green areas correspond to indium, oxygen, and silicon, respectively. The voids are again clearly revealed and might originate from fabrication residues or the FIB sample preparation. Figure 3d shows the In-Si heterointerface of the seed region. The interface is smooth and no indication of an interfacial layer such as from surface oxidation was observed. Both sides are single crystalline although there is no epitaxial relation between substrate and deposit. Indium crystalizes in body-centred tetragonal crystal structure whereas silicon in the face-centred diamond-cubic crystal structure. An epitaxial relation between deposit and the highly lattice mismatched substrate is therefore not expected. The lattice resolved image (Fig. 3e) shows that the entire cross section of the In deposit is one single crystal. This also suggests that the electrodeposited In is of high purity.

The results demonstrate that solution ED process results in high-purity, and crystalline indium deposits. Applying the ED process to selective growth in templates allowed to fabricate well-defined sub-micron structures made from indium. Furthermore, the use of a small embedded working electrode in the templates resulted in In structures which are mostly isolated from the Si substrate, and therefore ideally suited for device fabrication.

### Metal to semiconductor conversion in templates

#### Indium to InAs

The three-dimensional confinement, high purity, and thermal stability of the In-filled microstructures can be used as a starting point for the fabrication of In-alloys or In-group-V semiconductors on silicon. From all the available choices the focus here is on semiconductors, and arsenic was selected to saturate the electrodeposited indium to grow InAs from the Si seed.

**Fig. 4:**
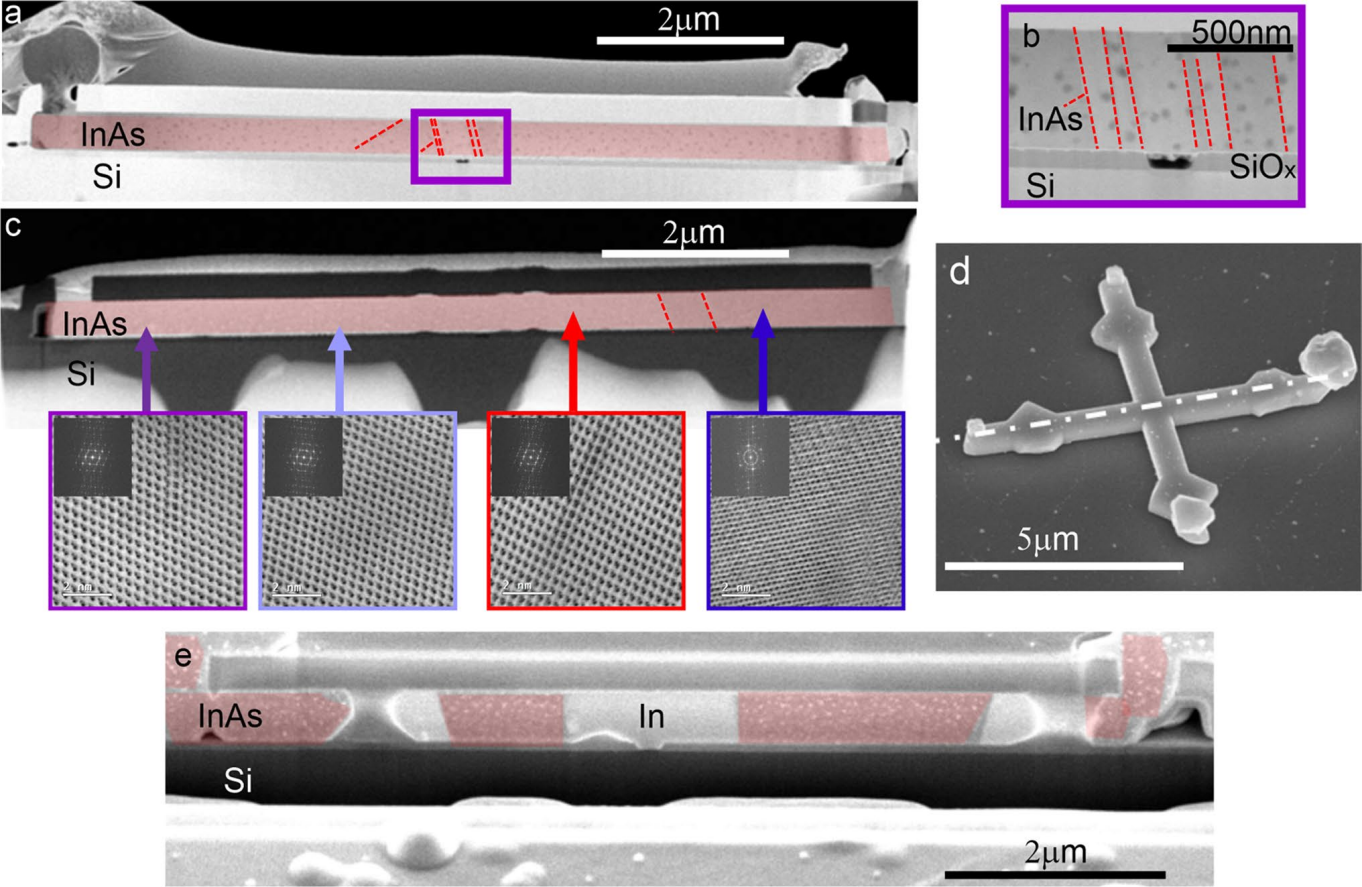
Analysis of In electrodeposit saturated with As. **a** Cross-sectional image of a template filled with InAs. **b** Close-up of the central part, showing a void in the seed area. **c** BF TEM overview of an InAs sample and corresponding high-resolution lattice images. **d** SEM tilted view of an InAs cross where the SiO_x_ template was removed. **e** SEM cross-sectional image of a device showing an InAs-In phase separation and large voids. InAs sections are false coloured for better visibility. Red dotted lines in (**a**–**c**) mark planar defects in the InAs crystal

The templates filled with electroplated In were loaded in an MOCVD reactor and heated well beyond the cracking temperature of TBAs (for more details please refer to the Experimental section). Here, similarly to [10, 11, 16] the As diffuses into the confined In melt and as in Viazmitinov et al. [20] is expected to epitaxially nucleate at the Si interface to form an InAs crystal, which will eventually expand as long as the supply of As is maintained, consuming all In. Figure 4d shows a representative result of a cross-structure (as in Fig. 3b, d) but after removal of the SiO_x_ template to better reveal the resulting InAs device. The crystalized InAs structure is overall well defined with some of the eight recessed edges structures sometimes missing, as observed for the initial In-filled structures. Also visible are larger deposits which extend beyond the templates on the ends of the cross, above the location of the template inlet holes. The structure of the InAs samples were assessed by fabricating cross-sectional samples and TEM lamellas as indicated in Fig. 4, followed by (S)TEM analysis. Figure 4a shows a low-resolution cross-sectional image with the InAs (false-coloured, red) surrounded by the template, inlet holes on either end of the template, and the seed hole at the centre of the device. Higher magnification of the central part is shown in Fig. 4b. Here, clearly resolved is a black area which corresponds to the location of the seed hole and indicates the presence of a void (bright field image) and missing material. The specific contrast observed in the InAs part is an indication of a crystalline structure and the presence of planar defects (marked with red dotted lines in Figs. 4a–c). The presence of a void and therefore loss of direct contact of the InAs with the Si substrate was observed in several samples. This suggests that with the process conditions used, initial InAs nucleation preferentially occurs in the melt or on the template surface, as opposed to the anticipated epitaxial nucleation on the crystalline Si surface.

(S)TEM analysis of another InAs device is shown in Fig. 4c (dark field image). Here, the position of the cross section is slightly off centre, beyond the location of the seed hole. As in the previous sample, the presence of planar defects running across the sample is visible in the low-resolution image. The corresponding lattice images and Fast Fourier transforms (FFTs) along the length of the device are shown in the high-resolution images below. The lattice information indicates that most of the sample is single-crystalline InAs with the presence of planar defects, while the rightmost part consists of nano-sized InAs crystals. A comparison of the experimental FFTs with simulation confirms the (111) InAs crystal orientation of the sample and the absence of an epitaxial relationship with the Si (001) substrate. We also observed samples that show non-uniform material contrast after As exposure (Figure S1). A SEM cross-sectional image of a non-uniform sample is shown in Fig. 4e. Here, InAs crystals are present on both inlet holes, followed by voids and segments of In and InAs with the In and InAs phase well separated. The seed area remains filled with In and interfaces with the Si substrate. These non-uniform process results are likely a consequence of multiple random nucleation events of InAs crystals in the melt or on the template surface. As soon as expanding InAs crystals block portions of the In-filled channel, the supply of As is ceasing, leaving a phase-separated InAs-In solid melt behind. While the occurrence of voids is observed, In leakage through the template walls could not be detected.

#### Indium to InSb

**Fig. 5:**
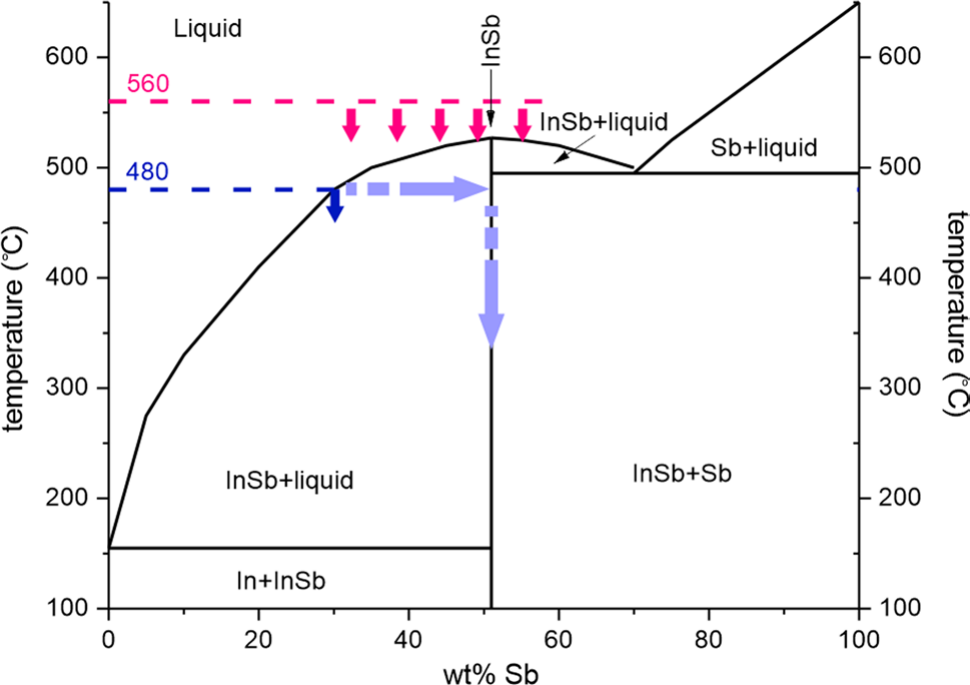
Phase diagram for In-Sb system with the two possible process paths for InSb solidification. Blue arrows indicate the low-temperature process leading to direct InSb nucleation. Light blue arrows depict continued saturation until stoichiometric composition, consuming all In. Pink arrows present the high-temperature path, where InSb crystallization is initiated by cooling of the melt

Analogous to the saturation and solidification experiments of the confined In melt with As, in-diffusion and crystallization can also be performed with antimony. Using antimony represents a unique system compared to other group-V elements (N, P, As) to form a compound semiconductor, with a low melting temperature of *T*_*m*__*InSb*_ = 527 °C. This allows for two distinct processes. The first process is similar to the previous case with InAs, but here the In melt is exposed to Sb and nucleation of the compound proceeds within the liquid In rich phase, ideally on the energetically more favourable Si seed surface. This process corresponds to the blue line in the phase diagram shown in Fig. 5.

The second process path possible with Sb is shown by the pink arrows in Fig. 5. Here, the temperature is initially set above the melting temperature of InSb. Working above the melting temperature of the semiconductor allows adjusting the melt composition before any nucleation and solidification occurs. Crystallization of the ideally stochiometric melt can subsequently be initiated by lowering the melt temperature. Compared to the As diffusion process which is self-terminating upon conversion of all the indium to InAs due to the efficient desorption of the As, too high (low) Sb exposure will result in an Sb-rich (deficient) melt, respectively, leading to a remaining Sb (In) phase besides the InSb. Thus, exact dosage of Sb is important if only the InSb phase is targeted.

##### Low-temperature process

**Fig. 6:**
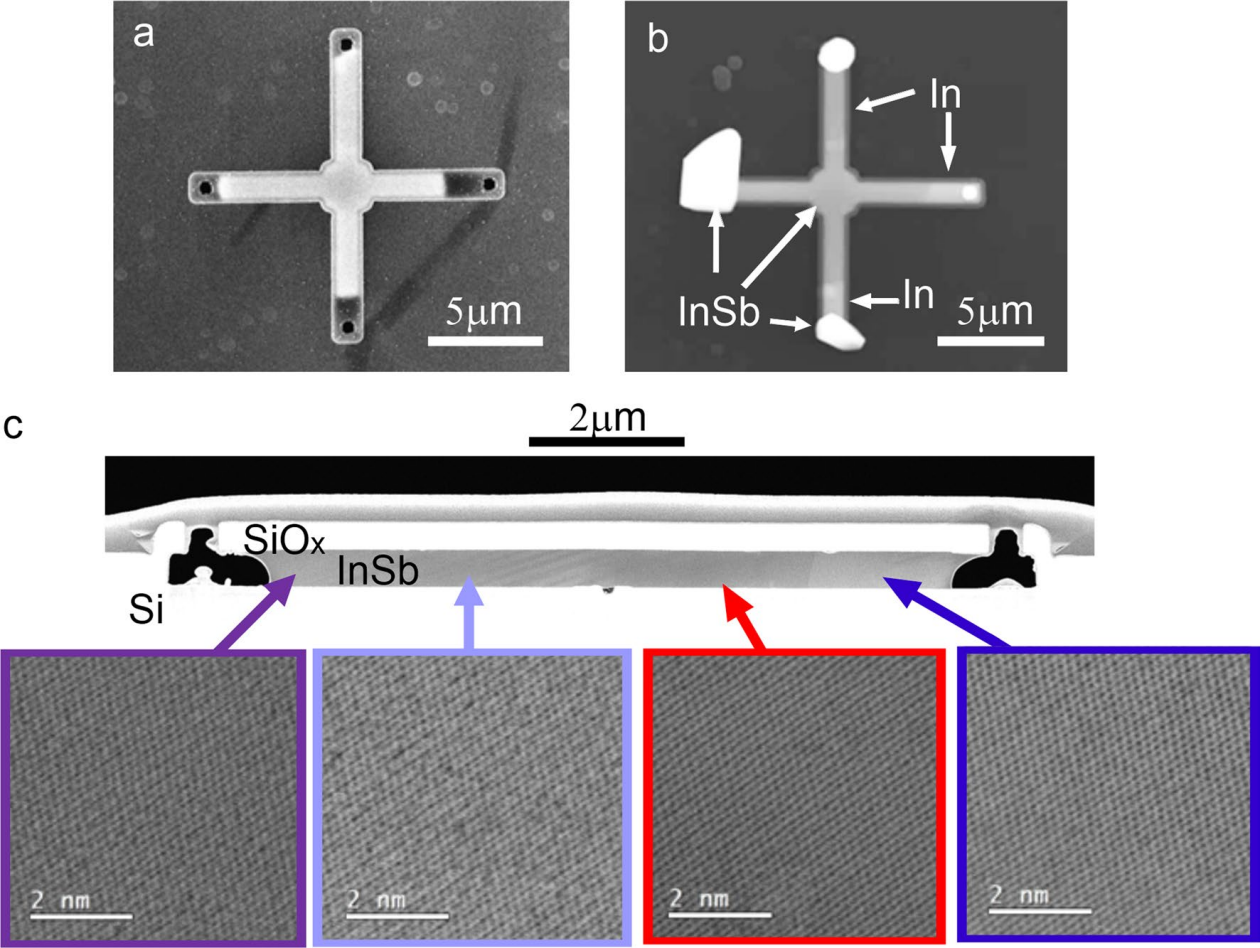
Analysis of In electrodeposit saturated with Sb at 480 °C. **a** Top-view SEM image showing uniform contrast of the InSb cross-structure. **b** A structure with clogged channels, revealing segments of In (bright) and InSb (darker) intensity. **c** BF-TEM overview of an InSb sample and corresponding high-resolution lattice images

The process commences with templates filled with electrodeposited In as described above and shown in Fig. 2. The samples were loaded in an MOCVD reactor and heated beyond the cracking temperature of the TMSb. The Sb flux and process duration were set to convert the In-filled channels into InSb, resulting in a structure of uniform contrast as evaluated by SEM inspection and shown in Fig. 6a. Too short (long) Sb exposure leads to remaining metallic In (Sb) deposits according to the In-Sb phase diagram and are thus detectable as areas of bright contrast in the SEM (Fig. 6b, Figure S2). Similarly to the As diffusion experiments, Sb-diffused structures with non-uniform contrast are observed. The non-uniform crystallization results are attributed to random nucleation events of InSb crystals in the melt or on the template surface as suggested for InAs (Fig. 5e). The crystal structure of low-temperature InSb was assessed by fabricating cross-sectional samples and TEM lamellas followed by (S)TEM analysis. Figure 6c shows a cross section of the low-temperature InSb sample together with high-resolution images taken from the spots marked with arrows. The overview image shows an 8 µm long InSb slab with the seed region in the middle being empty. The void between the Si seed and the InSb indicates that the initial nucleation did not occur on the Si surface, but rather randomly within the template, similarly to the observation for the InAs samples. High-resolution images were taken at several different locations along the lamella and reveal that the InSb sample is single-crystalline. During the preparation of the lamellas for the TEM investigation, the amorphization/surface modification of InSb samples was observed. This effect is known and was described in [21] for In-based III-V semiconductors (InP, InAs). Even with low-energy milling, some surface damage was observed (blurry spots on HR-TEM images in Fig. 6c), therefore, to extract crystal information, FFT was performed on HR-TEM images. The crystal orientation was (220) and no grain boundaries or dislocation were detected in the sample.

##### High-temperature process

The high temperature process significantly differentiates from the low temperature process as explained above. The In melt is enriched with Sb at a temperature above the melting point of InSb (560 °C), leading to a situation similar to the RMG process, were an ideally stochiometric melt is cooled and crystallized with the help of a seed surface. Here, the liquid InSb is expected to form a eutectic melt by the dissolution of Si from the seed area, with the eutectic InSbSi melt further lowering the solidification temperature. Finally, cooling of the melt will lead to a Si-doped InSb crystal, with all the Si above the equilibrium solubility crystallizing out as solid phase as observed for InGaAs-Si system [22].

**Fig. 7:**
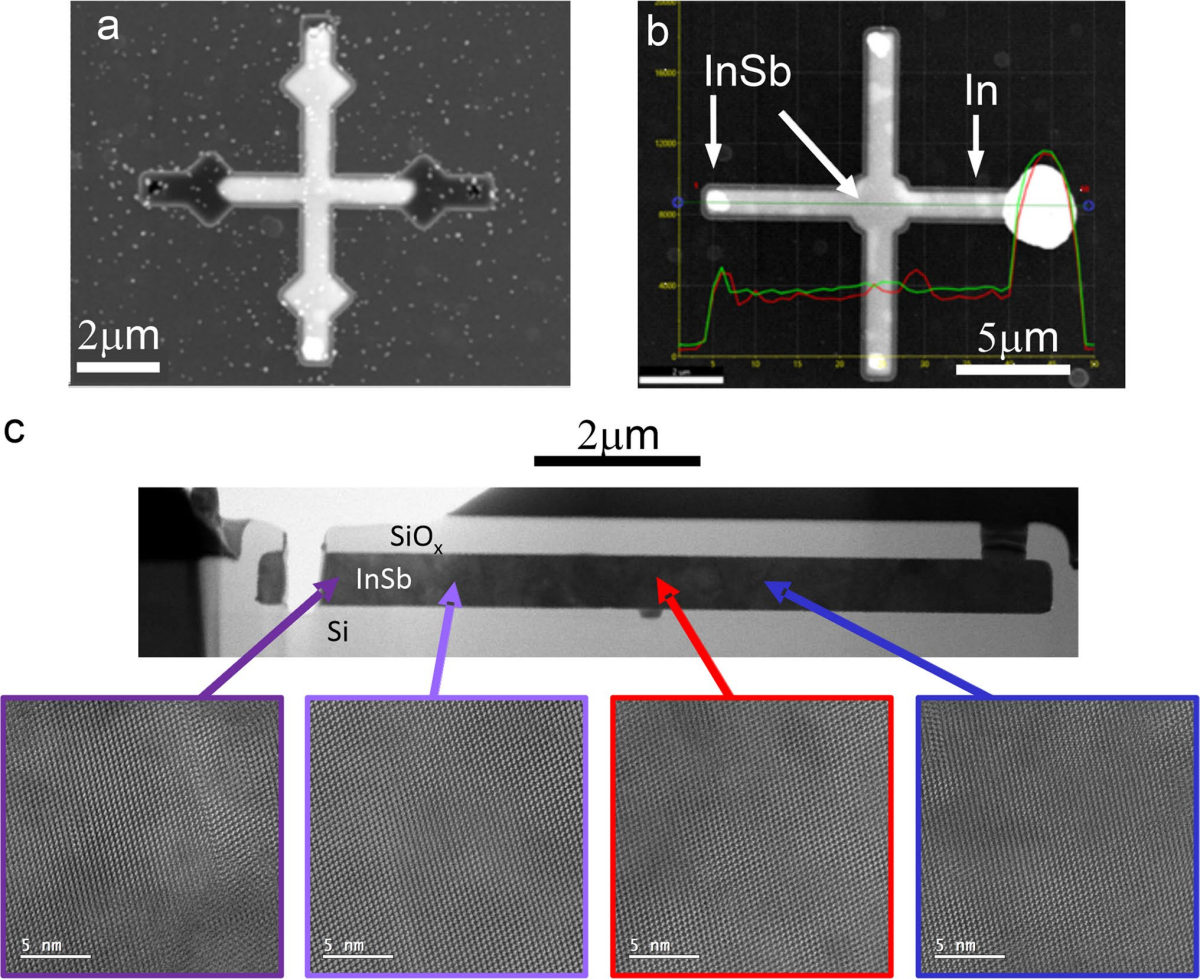
Analysis of In electrodeposit saturated with antimony above the InSb melting temperature. **a** Top-view SEM image showing uniform contrast of the InSb. **b** Example of a non-uniform sample, revealing segments of In (bright) and InSb (darker) with different contrast. **c** BF-TEM overview of an InSb sample cross section and corresponding high-resolution lattice images

The results of the high temperature process are summarized in Fig. 7. Figure 7a shows a template previously filled with In and converted to InSb. The image contrast as evaluated by SEM inspection is uniform and indicates complete conversion of In to InSb. This is supported by EDX mapping of In and Sb signal (Figure S2) showing a uniform concentration. Samples with non-ideal solidification of In-Sb melt were also observed as evaluated from contrast variations in the SEM and EDX line scans signals (Fig. 7b). Here, randomly distributed In inclusions are observed, indicating uncontrolled nucleation.

For the structural characterization, an InSb sample of uniform appearance was selected for TEM investigations. An overview image of the prepared InSb sample is shown in Fig. 7c. High resolution TEM of several selected areas show a single crystalline structure with (220) orientation, and the absence of dislocations or grain boundaries. Interestingly, the InSb appears to be in contact with the Si surface. However, there is no epitaxial relationship between the Si and the InSb, and the expected dissolution or etching at the Si interface as previously reported for InSb-Si system [6] by the InSb melt was not observed. Both findings suggest that an unintentional contamination of interface was introduced during the saturation process, suppressing the formation of a heteroepitaxial InSb-Si interface.

## Discussion and conclusions

Electrochemical deposition of indium in lithographically structured, hollow templates was studied. The deposits nucleated on a small, exposed Si area within the silicon oxide template structure, and filled the template as single crystalline deposit. The In-filled templates were temperature stable up to 560 °C, and In recrystallized into single crystals after cooling. The low solubility (< 0.1%) of Si in In [23] limits the dissolution of Si into In and the formation of etch pits in the seed area, in accordance with the TEM observations showing a smooth In-Si heterointerface.

Synthesis of InAs and InSb was achieved by exposing the liquified In in the template to As and Sb vapours. The diffusion and crystallization process yielded similar results for InAs and InSb. However, unexpectedly the semiconductor-Si contact was not present anymore. This finding indicates that the III-V (epitaxial) nucleation of the melt on the Si seed surface is not favourable within the experimental conditions studied, and that nucleation on the template surface dominated. The occurrence of heterogeneous nucleation events is further supported by the observation of semiconductor (InAs, InSb) segments sometimes clogging the template channels and leading to trapped In and voids. The preferential nucleation of the III-V melt on the silicon oxide surface is surprising given that crystallization experiments using similar settings were reported in zone melt recrystallization [24] and RMG [25] which however used much larger nucleation areas. Random or heterogeneous nucleation was also observed in [18] and attributed to unintentional interfacial layer that isolates the semiconductor from the Si substrate. Thus, increasing the Si nucleation area and improving the melt-Si interface are promising next steps towards efficient nucleation and heteroepitaxial growth.

In summary, we have demonstrated selective electrodeposition of a group-III metal and the vapour phase diffusion of group-V element in lithographically defined templates to integrate III-V semiconductors on silicon. The process was exemplified by synthesizing InAs and InSb and resulted in single crystalline material. With improved template fabrication, selective nucleation on Si and heteroepitaxial integration of InSb and other semiconductors and alloys should become possible using the demonstrated two-step process and thus opening new possibilities of fabrication of complex micro and nanodevices directly on silicon substrates.

## Experimental

Highly doped p-type silicon wafers (100) were used as a global working electrode and starting point for the fabrication of SiO_x_ templates. A 100 nm thick SiO_x_ layer was deposited on the wafer using atomic layer deposition (ALD). Subsequently, holes with a diameter of 100 nm were etched into the SiO_x_ using reactive ion etching (RIE) with Ar and CHF_3_ to define the electrode/seed area. Then, a seed protection layer of 10 nm Al_2_O_3_ was introduced by ALD followed by a 300 nm thick sacrificial amorphous Si (α-Si) layer obtained via sputtering. Subsequently, the α-Si layer was patterned with the layout of template structures, using a SiO_x_ hard mask and etched using HBr-based inductively coupled plasma (ICP) etching, and coated with a 300 nm thick SiO_x_ shell using ALD and plasma-enhanced chemical vapour deposition (PECVD). Finally, inlets were patterned on the template structures and etched into the SiO_x_ shell, exposing the α-Si layer. The sacrificial α-Si layer was etched away with XeF_2_, resulting in hollow template structures. We note that a low density of residues from the etch processes are visible as round features across the wafer outside the templates (Fig. 2) which could not be removed. The as-prepared substrate was then dipped in a buffered HF solution to dissolve the Al_2_O_3_ protection layer and diluted (2.5%) HF was used to remove the native oxide on the exposed Si surfaces within the channels before electrodeposition.

In order to minimize the possibility of introducing unwanted elements into the samples the ED process was designed in a way to reduce the presence of impurity sources. For that reason, deoxidized electrolyte containing only DI water and inorganic indium salt was used. For the hardware (electrodes and electrochemical cell) a cleaning procedure before every single ED process was applied. A working electrodes (WEs) (Si chips with 3D SiO_x_ templates) were cleaned in HF/DI water and isopropanol right before the electrodeposition (less than 45 s between cleaning/drying and mounting WE in ED cell). 99.999% Pt counter-electrode (CE) was soaked in diluted HNO_3_ and then DI water prior to electrodeposition. The In electrodeposition solution contained 0.2 M InCl_3_ (Sigma-Aldrich, > 98%) salt in DI water with pH adjusted to 2.4 by the addition of HCl and was bubbled with Ar prior to and during the synthesis process. A 2-electrode galvanostatic technique was chosen to have maximum control over the homogeneity of electrodeposit. The electrodeposition experiments were carried out using the potentiostat (BioLogic SP300) with two electrodes (WE and CE) mounted at a distance of 1 cm. All electrodeposition experiments were performed at room temperature. Based on preliminary studies current density of − 10 mA/cm^2^ and electrodeposition time of 240 s were chosen for In deposition inside templates (active surface area ~ 0.0722 cm^2^).

After electrodeposition, the templates were placed in the MOCVD chamber to saturate the indium with group V element. For saturation with arsenic a tertiarybutylarsine (TBAs) precursor at 580 °C and with antimony a trimethylantimony (TMSb) precursor at 480 °C or 560 °C, (Dockweiler Chemicals) were used. The cooling rates were 10 °C/min and 50 °C/min. The total saturation time for the V element was 45 min for all samples. After annealing, the SiO_x_ capping layer was removed using a combination of reactive ion etching and HF etching (either wet or vapour), and the morphology, crystallinity, and chemical composition were characterized using scanning electron microscopy (SEM, Hitachi SU8000) and analytical (scanning) transmission electron microscope ((S)TEM, JEOL ARM200F) equipped with energy-dispersive X-ray spectroscopy detector. Lamellas for (S)TEM–EDX analysis were prepared using the focus ion beam technique (Dual-beam FIB/SEM, FEI Helios NanoLab 450S). We note that the InSb sample preparation was sensitive to Ga ion beam exposure, leading to significant surface damage, reduced TEM image quality, and even melting of the InSb during FIB processing.

## Supplementary Information

Supplementary materials (DOCX 570 KB)

## Data Availability

Not applicable.
