# Supplementary material for: Selective electrodeposition of indium microstructures on silicon and their conversion into InAs and InSb semiconductors
Source: Discov Nano. 2023 Feb 7;18(1):4. doi: 10.1186/s11671-023-03778-9 (PMC9902586; doi:10.1186/s11671-023-03778-9)
Supplement: Supplementary file 1 — Supplementary materials (DOCX 570 KB) [file 11671_2023_3778_MOESM1_ESM.docx]

**Selective electrodeposition of indium microstructures on silicon and their conversion into InAs and InSb semiconductors**

Katarzyna E. Hnida-Gut^1,2,*^ Marilyne Sousa^1^, Preksha Tiwari^1,3^, Heinz Schmid^1^

^1^IBM Research Europe – Zurich, Zurich, Switzerland.

^2^Now at: IHP Leibniz Institute for High Performance Microelektronics – Frankfurt (Oder), Germany.

^3^Now at: Polariton Technologies Ltd., Zurich, Switzerland.

*corresponding author: hnida@ihp-microelectronics.com

Keywords: integration, saturation, electrodeposition, recrystallization, III-Vs, TASE


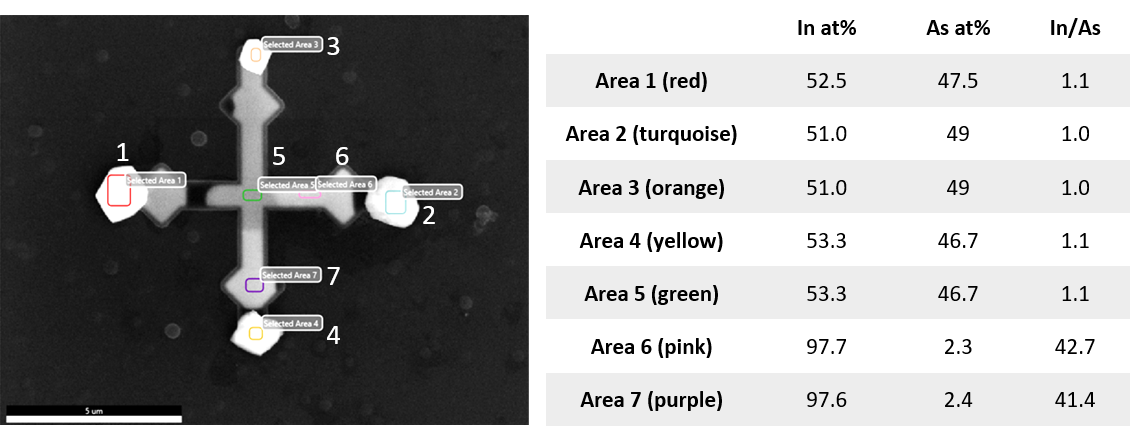


**Figure S1.** SEM image of template filled with In-InAs material with marked areas selected for EDX analysis together with SEM-EDX point analysis.


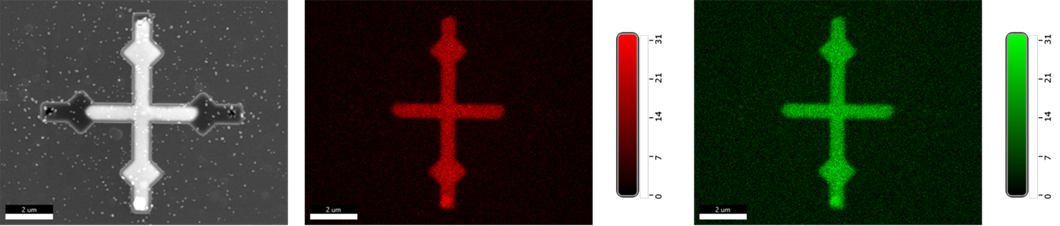


**Figure S2.** SEM image of the sample after the high-temperature process. Sb-saturated sample together with In and Sb EDX distribution maps showing uniform composition.
